# Contextual F0 cues can outweigh talker F0 cues in fricative perception

**DOI:** 10.3758/s13414-026-03246-3

**Published:** 2026-05-19

**Authors:** Orhun Uluşahin, Hans Rutger Bosker, Antje S. Meyer, James M. McQueen

**Affiliations:** 1 Max Planck Institute for Psycholinguistics, Wundtlaan 1, 6525 XD Nijmegen, The Netherlands; 2 Donders Centre for Cognition, Radboud University, Thomas Van Aquinostraat 4, 6525GD Nijmegen, The Netherlands

**Keywords:** Speech perception, Talker familiarity, Spectral contrast effects, Fricative perception, Cue integration

## Abstract

Listeners use information in the speech signal as well as linguistic and real-world knowledge to tackle the immense variability of speech. Here, we focus on the use of contextual and talker-bound fundamental frequency (F0) cues during the perception of voiceless fricatives’ center of gravity (CoG). In Experiment 1, Dutch participants heard the sentence *Nu komt het woord ?ok* (“Now comes the word ?ok”) where “?” denotes a fricative from a synthetic /s-ʃ/ continuum in three carrier F0 conditions (low, mid, high), and indicated whether they heard the high-CoG /sɔk/ “sock” or the low-CoG /ʃɔk/ “(to) trudge.” We found a contrastive effect of context F0 on CoG perception whereby hearing a high F0 carrier sentence led to a lower fricative CoG perception. In Experiments 2a and b, an exposure phase was added where participants either heard a low or a high F0-shifted talker. At test, all participants heard fixed-F0 words /?ɔk/ and indicated whether they heard *sok* or *sjok*. Talker F0 guided participants’ responses in both experiments, but across more trials in Experiment 2b than in Experiment 2a. In Experiment 3, combining context and talker manipulations, two groups of participants heard either a low or a high F0 talker in exposure, and both low and high F0-shifted carrier sentences at test. There was a large context F0 effect, but crucially, no talker F0 effect. Overall, we found evidence that both contextual and talker-bound F0 cues have contrastive effects on fricative perception, and that contextual cues, when present and sufficiently reliable, can outweigh talker cues.

## Introduction

Human speech is infinitely variable. Anything from a talker’s mood to the environment in which they happen to be talking can affect the speech signals we encounter as listeners. In addition to the variation in the context in which speech occurs, different talkers also vary across a large number of acoustic features of their speech, such as how loud particular sounds in their speech are, how fast they talk, and how high the pitch of their voice is (Johnson & Sjerps, 2021). Despite this immense variability, speech perception remains quick and effortless in most daily interactions. This leads to one of the central questions of speech perception research: How does the brain adjust to infinite variation? Here, we focus on how listeners track talker pitch, how they use this information in subsequent fricative perception, and how knowledge of a talker’s typical pitch interacts with the acoustic information present in incoming speech.

The brain partly tackles variability by rapidly adjusting to acoustic variation in incoming speech. The adjustments that strictly rely on the acoustic information present in the incoming signal (i.e., the acoustic context) are commonly referred to as being “signal-driven” (i.e., as opposed to being influenced by higher-order structures or sources of information; Martin, 2016). Multiple lines of evidence highlight the susceptibility of the perception of individual phonemes to the surrounding context. For instance, when a particular vowel (especially a temporally ambiguous one) is preceded by a sentence with a fast speech rate, it is perceived as being longer than when it is preceded by a sentence with a slower speech rate (Kidd, 1989; Miller & Dexter, 1988; Reinisch & Sjerps, 2013; Reinisch, 2016a). Manipulating the F1 and F2 of a perceptual target’s context can affect the subsequent perception of vowel quality (Ladefoged & Broadbent, 1957), and even exposure to non-speech can influence the perception of speech sounds (Laing et al., 2012). Notably, such effects often rely on contrasts in specific acoustic dimensions. In the spectral domain, for example, hearing a high F0 stimulus before a Mandarin tone biases its perception towards a low tone, while hearing a low F0 precursor leads to a high tone perception, as listeners dynamically adjust their reference F0 values to which tones are relativized (Moore & Jongman, 1997). Similarly, if a harmonic spectrum with vowel-like peaks precedes a flat harmonic spectrum, the flat spectrum is more likely to be perceived as a vowel (i.e., despite not having a typical vowel-like spectrum) due to the “popping out” of the spectral peaks in subsequent perception (Summerfield et al., 1987). Thus, the successful disambiguation of an incoming signal often depends on the perceptual system’s evaluation of the differences between the target sound and the acoustic context (Stilp, 2020).

In addition, the brain relies on (meta)linguistic or real-world knowledge to aid in speech perception. In contrast to signal-driven processes, these constitute a “knowledge-driven” influence on the perceptual stream. For instance, listeners store information about different talkers on a talker-by-talker basis (Cummings & Theodore, 2022; Kleinschmidt, 2019; Kraljic & Samuel, 2005; Nygaard et al., 1994; Theodore & Monto, 2019), which provides an array of perceptual benefits for speech signals coming from familiar talkers, such as better speech detection in noise (E. Holmes et al., 2018; Nygaard et al., 1994) and more efficient word recognition (Nygaard & Pisoni, 1998). Some empirical work suggests that these perceptual benefits can be obtained with as little as 10 min of exposure to unfamiliar speech (E. Holmes et al., 2021) and are present even when a listener is not able to explicitly report a voice as being familiar (E. Holmes et al., 2018). Thus, while signal-driven processes may underlie adjustments to speech in context, dynamic representations of individual talkers’ speech allow us to adjust to differences between talkers that are stable over longer periods of time. These talker representations are thought to be flexible to the extent that they can be updated through consistent novel input (Kleinschmidt & Jaeger, 2015), while also affording abstractions that allow listeners to group different talkers into groups based on similarity (e.g., dialect, accent, speech impairment) (e.g., Bradlow & Bent, 2008).

Among the acoustic differences between talkers that are attributable to articulatory anatomy, the largest are primarily functions of age and sex. The role of voice fundamental frequency (F0) as a strong indicator of sexual dimorphism as well as its ubiquity in speech has led to its extensive study, both in biology and in psycholinguistics (Pisanski et al., 2021), providing insights into its production and perception. Here, we also focus on the perception of F0, and its psychoacoustic counterpart, pitch. However, it is worth mentioning that the present study is not concerned with the role of F0 in indicating talker sex, but with within-talker (and thus within-sex) variation, which cannot be accounted for solely in physiological terms.

Although F0 is the largest component and strongest acoustic indicator of pitch, listeners still perceive pitch when an incoming auditory signal contains no F0 information (e.g., in whispered speech). Additionally, previous psychoacoustic research has demonstrated that listeners are able to perceive an F0 by extracting information from the harmonics of signals that do not contain any energy at the perceived F0 (e.g., Lau et al., 2017; Mehta & Oxenham, 2022; Schouten, 1940). More specifically, in speech-perception research, Heeren (2015) used a two-interval forced-choice (2AFC) discrimination task in which native Dutch listeners had to indicate whether a vowel-consonant–vowel (VCV) stimulus that followed another VCV stimulus was higher or lower in pitch. The results indicated that voiceless fricatives (i.e., sounds involving frication only, without any involvement of the vocal folds and hence lacking F0) that were deliberately produced with a pitch target (i.e., high or low) were consistently identifiable as higher or lower in pitch by listeners, both in whispered (i.e., speech without F0) and in voiced speech. Other studies similarly indicated that listeners can successfully perceive lexical tone (Zhang et al., 2022) and intonation (J. N. Holmes & Stephens, 1983) in whispered speech. Thus, secondary (i.e., non-F0) correlates of pitch targets (e.g., duration, spectral skewness, larger variation in vowel formants) were used to perceive pitch in the absence of F0 as listeners adjusted to the co-variation of F0 and other acoustic cues in production. Here, we emphasize that this link between F0 and its acoustic co-variates is partly based on physiology, as indicated by greater acoustic similarity between monozygotic twins compared to dizygotic twins (Debruyne et al., 2002; Van Lierde et al., 2005), and electropalatographic evidence for the influence of palatal morphology on fricative CoG (Weirich & Fuchs, 2013; /s/–/ʃ/ data from German) as well as vowel quality (Fuchs et al., 2008). These studies indicate that physical characteristics of the articulatory system that affect the production of F0 can affect CoG, and vice versa.

The susceptibility of pitch perception to cues other than F0 presents multiple avenues for the measurement of perceptual adjustments to F0 using various acoustic correlates of pitch. One of these cues is the spectral center of gravity (CoG), which refers to the weighted average of the frequency components of a signal. Measures of CoG enable measurements of pitch correlates in sounds that do not contain F0, whereby a higher CoG correlates with a higher pitch percept. For instance, the (English) phoneme /s/ has a higher CoG than /ʃ/ (Jongman et al., 2000; Newman et al., 2001). Thus, within this framework, a fricative like /s/ has a higher perceived pitch than /ʃ/, despite the lack of an F0 in both signals. This study uses fricative perception as a measure of perceptual adjustments to F0 variation. This particular proxy measure has previously been used in psycholinguistic research on account of a contrastive link between F0 and fricative CoG due to the co-variation of these two acoustic features. For instance, Niebuhr (2017) found that F0 context modulated /ʃ/-/s/ phoneme identification through pitch perception. Niebuhr generated a 10-step /ʃ/-/s/ continuum with evenly spaced CoG. In a categorization task where minimal word pairs could only be distinguished through the disambiguation of the critical fricatives (e.g., /pas/-/paʃ/ “passport-doublet”), high F0 sentence contexts (i.e., rising F0 before the target word containing the fricative) produced a higher proportion of /ʃ/-word responses than /s/-word responses, and low F0 sentence contexts (i.e., falling F0 before the target word containing the fricative) produced a higher proportion of /s/-word responses than /ʃ/-word responses. Thus, F0 information in the phonological context interacted with the categorical perception of fricatives, with an inverse relation to fricative CoG (i.e., higher F0 biases towards lower CoG perception and lower F0 biases towards higher CoG perception), suggesting that pitch perception plays a direct role in the perception of the spectral characteristics of voiceless fricatives. Note that we distinguish between the perception of a fundamental in a given segment and the perception of pitch correlates including but not limited to the perception of a frequency distribution in a voiceless fricative. Throughout this article, we use “perceived (fricative) pitch” to refer to the perceived characteristics of fricatives, “F0” to refer to fundamental frequency, and “pitch” to refer the perceptual counterpart.

A signal-driven process may explain part of this contrastive effect. Specifically, the co-variation of F0 and fricative CoG in production could encourage listeners to normalize incoming speech signals such that the perception of either cue would be directly tied to pitch perception. This would in turn manifest in perceptual mechanisms whereby the pitch information in the context is regressed out from the pitch perceived on the target fricatives. In more specific terms, if the perceptual system expects a high CoG fricative because the preceding context contains high F0 information, the perception of a target fricative is dependent on the subtraction of the *observed* fricative CoG from the *expected* CoG, leading to a contrastive influence on the perception of the target (McMurray & Jongman, 2011). Consequently, a greater distance from the expected value may result in a larger contrastive effect in more ambiguous targets (e.g., fricatives in Niebuhr, 2017).

In addition to this signal-driven mechanism, previous research has also revealed various knowledge-driven influences on fricative perception. For instance, listeners are more likely to categorize an ambiguous fricative between/s/and/ʃ/as/s/when it is embedded in a male voice than when it is embedded in a female voice. This effect is also observed when talker sex is cued by vocal tract size and even by images of talkers alone (Munson et al., 2017; Strand & Johnson, 1996). This effect is further modulated by factors as seemingly unrelated to speech perception as a given talker’s perceived sexual orientation (Munson et al., 2006). Thus, fricative perception can also be guided merely by adjustments to how a listener believes a talker might sound in accordance with what they know about the talker, including their speech.

While these different lines of evidence attribute the same effects to different sources, the direction of the effect (i.e., the inverse correlation between F0 and fricative CoG) remains consistently contrastive. Thus, the listener consistently anticipates and adjusts for the idiosyncrasies of a talker’s speech. However, these accounts largely rely on the immediate acoustic context or adjustments to supercategories of talkers (i.e., adjustments to “female talkers” rather than “this particular female talker,” given the common multiple-talker designs). Less work has been done on the interplay between acoustic context and the use of knowledge about individual talkers. The limited number of existing studies typically report interactions of local (i.e., contextual) and habitual (i.e., talker-bound) variation, with various constraints that shape those interactions. For instance, a talker’s habitual speech rate seems to have a contrastive effect on the perception of vowel duration (i.e., a temporally ambiguous vowel sounds longer in fast speech), but only if the talker’s speech rate was consistent (Maslowski et al., 2019). Similarly, after exposure with a habitually fast versus slow talker, listeners only display a contrastive effect of this knowledge about a given talker’s habitual speech rate on vowel duration if target words are presented in isolation (i.e., without local contextual information; Reinisch, 2016b). These results suggest that utilization of talker-specific information may be gated by the quantity and variability of local contextual information. However, it is also worth noting that more recent studies (e.g., Ting & Kang, 2023) which used similar designs to investigate a possible contrastive effect of speech rate on voice onset time (VOT) perception of/p/-/b/contrasts have reported that talker effects might not be identifiable when a critical test contrast is absent from exposure material.

In addition, as recognized by Reinisch (2016a, 2016b), speech/articulation rate may not be a very robust indicator of talker identity as it tends to vary more within than between talkers (Quené, 2008; provides data specifically for Dutch). In contrast, F0 is a more reliable indicator of talker identity (Johnson, 1990) as it tends to vary less within talkers compared to speech rate (e.g., Pépiot, 2014). Investigations of the perceptual effects of talker-bound F0 information may therefore lead to different outcomes than studies which investigated the more highly variable (i.e., within-talker) speech rate.

In the present study, we describe three experiments which further investigate the link between F0 and fricative perception, both in the proximal acoustic context (i.e., preceding sentence mean F0) and in talker-bound perceptual adjustments (i.e., knowledge of a talker’s mean F0) to test whether the F0-CoG contrast effect can also be guided by talker knowledge. These experiments were designed with two aims. First, we sought to establish whether fricative CoG perception is a valid proxy measure for both signal-driven and knowledge-driven perceptual adjustments to F0 variation. We thus aimed to shed light on whether listeners use talker F0 knowledge as well as F0 information in the contextual signal to adjust to incoming speech. Second, if so, we asked how these cues are weighed or integrated in perception. By using a within-talker (and thus within-gender) paradigm, we sought to reveal fine detail regarding the relative contributions of phonological context and talker information on fricative perception. In turn, these findings will inform theories of how these cues are weighed in the perceptual system, with implications for real-life interactions where some talker information is often present alongside the acoustic content of a speech signal.

As the first step in our investigation, in Experiment 1, we tested whether perceptual categorization of synthesized fricatives on a (Dutch) /s/-/ʃ/ continuum was influenced by F0 context within one and the same talker and was thus a reliable proxy measure for adjustments in pitch perception. After establishing the validity of the CoG proxy measure, we then conducted another three experiments to investigate the role of talker F0 information (Experiments 2a and 2b) and its interaction with contextual F0 information (Experiment 3).

## Experiment 1

Experiment 1 used tokens from a synthesized eight-step fricative continuum between /s/ and /ʃ/ in the carrier sentence *Nu komt het woord /?/ok* (“Now comes the word /?/ok”) (where /?/ denotes a synthesized fricative). Participants had to categorize the word as *sok* (“sock”) (/sɔk/) or *sjok* (“to trudge”) (/ʃɔk/). The carrier sentence itself had three F0 context conditions: Low, mid, and high.

We had one primary hypothesis: We expected the F0 information in the carrier sentence to have a contrastive influence on fricative CoG perception. In addition, our design had two features that translated into our two secondary hypotheses: We expected the synthesized fricative continuum to induce a smaller proportion of /s/ responses over continuum steps going from /s/ to /ʃ/ and we predicted that a potential F0 context effect would be largest for the more ambiguous (i.e., middle) steps of the continuum.

### Method

#### Participants

Ten healthy adult female native speakers of Northern Standard Dutch (age range = 18–29, *M*_*age*_ = 24, *SD*_*age*_ = 2.56 years) participated in the experiment. They self-reported through the Max Planck Institute participant database as having no hearing or speech impairments and having normal or corrected-to-normal vision. The sample was restricted to female participants primarily to ensure minimum differences in the productive F0 ranges of the participants. Participants gave informed consent as approved by the Ethics Committee of the Faculty of Social Sciences of Radboud University (project code: ECSW-2019–019).

#### Materials

##### Recordings

Our primary aim in stimulus design was to have precise control over a synthesized fricative continuum modeled after a female native Dutch speaker’s natural pronunciation of the Dutch fricatives /s/ and /ʃ/. To ensure that our stimuli would reflect naturalistic intensity contours, durations, and spectral profiles as well as some coarticulation, we recorded from a single female speaker the Dutch words “sok” and “sjok”’ at the end of the carrier sentence “Nu komt het woord…” (“Now comes the word…”), which itself contained no other fricatives (the speaker elided the /h/ in *het*).

##### Creating the fricative continuum

**Fig. 1:**
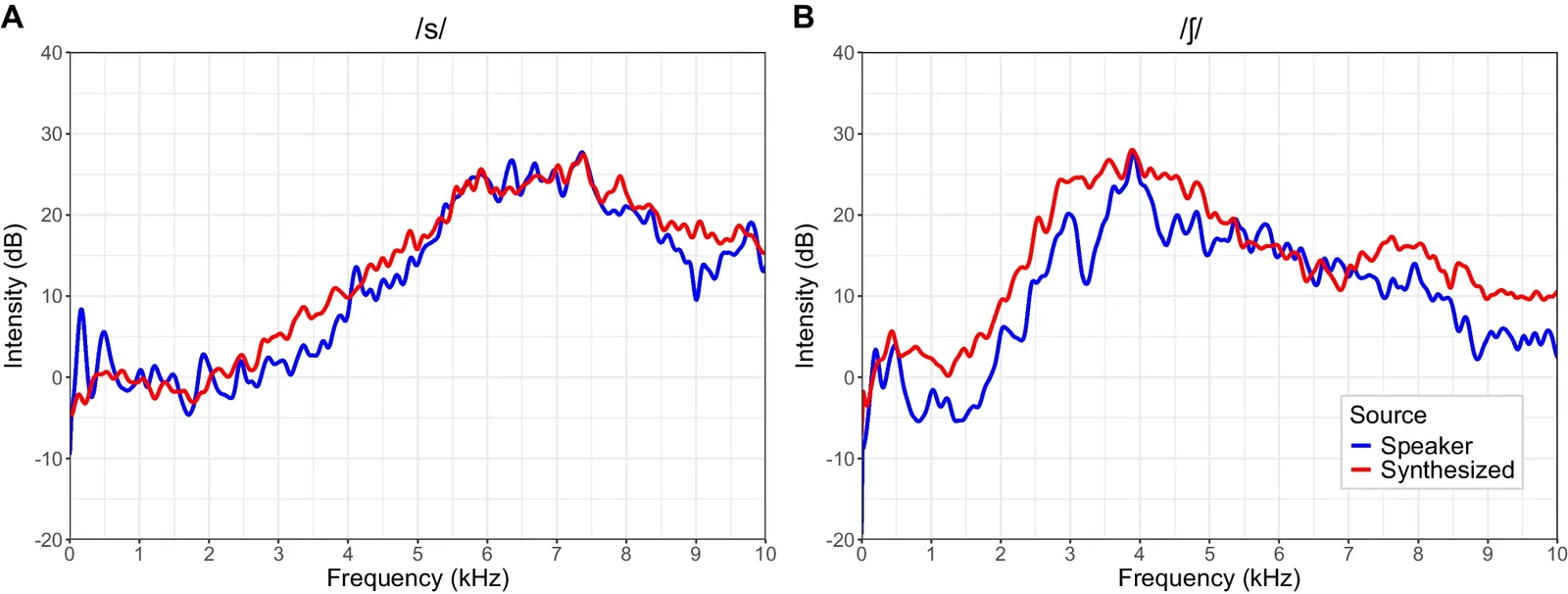
Spectra of synthesized fricatives (below 10 kHz). Plots show comparisons of the spectra (below 10 kHz) of the reference speaker’s /s/ (Fig. 1 A) and /ʃ/ (Fig. 1B) tokens with the synthesized fricative continuum’s endpoints. The blue lines indicate the speaker’s fricative spectra, and the red lines indicate the corresponding endpoints (i.e., step 1 for /s/, step 8 for /ʃ/) from the synthesized continuum. The Y axis displays the phoneme intensity at frequency ranges visible on the X axis. A high degree of overlap can be seen in both figures. The rest of the fricative continuum (i.e., steps 2–7) was linearly interpolated from the acoustic features of the two synthesized endpoints (i.e., steps 1 and 2). The fricatives used across all experiments were from this continuum (see OSF for visualization of the entire continuum)

**Fig. 2:**
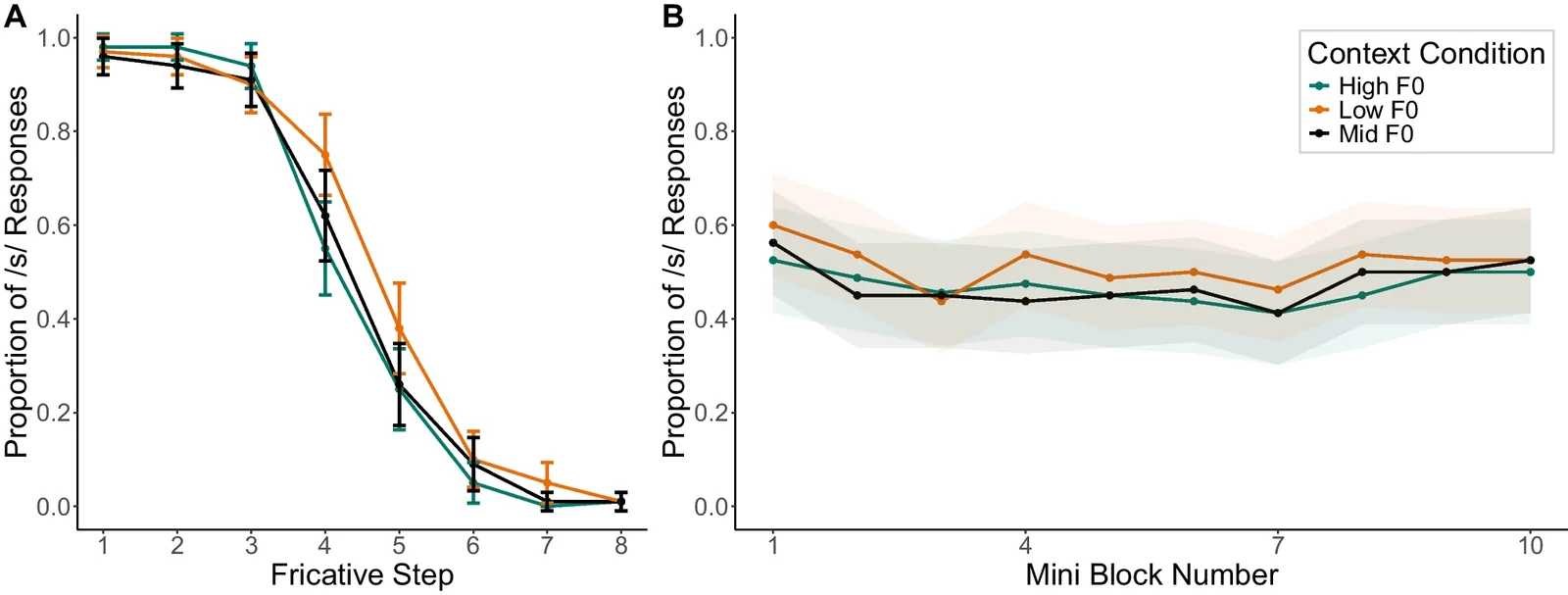
Response proportions by fricative step and mini-block across all participants per F0 context condition in Experiment 1. Figure 2 A shows the proportions of /s/ responses by fricative step and context (F0) condition, and Fig. 2B shows the proportions of /s/ responses across mini-blocks of 24 trials each for the three context conditions. Context conditions are indicated by line color (green = high F0, orange = low F0, black = mid F0). In Fig. 2 A, the error bars and ribbons indicate 95% confidence intervals. In Fig. 2 A, while the fricative continuum strongly predicts participants’ responses, larger differences in /s/ response proportions between the low and high F0 context conditions emerge in the more ambiguous steps of the synthesized /s/-/ʃ/ (i.e., high CoG-low CoG) continuum. In line with our predictions, this constitutes evidence that F0 information present in the acoustic context incurs a contrastive adjustment in fricative CoG perception whereby, for instance, hearing high F0 leads to lower CoG perception. At the same time, the mid F0 context condition consistently leads to response proportions between the high and low F0 context conditions. This, combined with the lack of significant variation across the experiment evident in Fig. 2B, indicates that participants’ responses were primarily guided by the context F0 manipulation implemented in Experiment 1. Note that, because the context effect is strongest for the ambiguous steps but the data are collapsed over all steps in Fig. 2B, there is no context effect visible in that figure.

We used a Praat script (a modified version of the script initially used by Winn et al., 2013; see Online Supplementary Material (OSM) on the Open Science Framework (OSF)) to create an artificial eight-step continuum, modelled on the spectral profiles of our speaker’s /s/ and /ʃ/, where step 1 was closely modelled on /s/ and step 8 on /ʃ/ (see Fig. 2A). The fricatives were synthesized by shaping white noise around five spectral peaks for each endpoint. For each peak, we specified a distinct center frequency, a distinct bandwidth, and a distinct relative amplitude with respect to the center frequency. The five peaks were then added together for each endpoint. It is worth mentioning that acoustic properties of the recordings that co-vary with CoG (e.g., spectral variance, skewness, and kurtosis) have also been manipulated in this process. Throughout this article, we use the phrase “fricative CoG” and its manipulations as a shorthand for “fricative CoG and its perceptual covariates.” The resulting fricatives had a fixed duration (194 ms), a fixed rise time, and a fixed fall time. (These fricatives are available on the OSF, and a summary of their CoG values is provided in “cogs.txt” under “Experiment 1, Stimuli “ alongside the speaker’s reference values). The intermittent steps (i.e., steps 2–7) were synthesized automatically (using linear interpolation) from values derived from input parameters for the two endpoints (i.e., steps 1 and 8) (Fig. 1).

To further increase experimental control over the stimuli, we used another script to compute a target-intensity profile based on the mean intensity contour of both fricatives from the original recordings and manipulated each step of the continuum to have this mean intensity profile (see OSF for reference recordings).

##### Creating the F0 context conditions

Unlike the fricative continuum, the carrier sentences were not synthesized but resynthesized from our speaker’s original recordings in one of three F0 conditions: low, mid, and high. The mean F0 of our speaker’s carrier sentence was 232 Hz (i.e., based on a sentence recording with the word *sjok*). We computed the F0 values in Hz for four semitones above (i.e., 293 Hz) and four semitones below (i.e., 184 Hz) this average as our targets for the high and low F0 context conditions, respectively. The four semitone differences were chosen based on the variance in F0 distributions observed in Dutch speakers (Adank et al., 2004). The specific formula we used to convert semitones into frequency measures (Hz) for manipulation in Praat was based on the definition that accepts the semitone formula as a logarithmic derivation of the Hertz scale (e.g., Nooteboom, 1997) where the distance between the frequencies f_1_ and f_2_ is given by the following equation:

$$D=12*{log}_{2}\left({f}_{1}/{f}_{2}\right)=12/{log}_{10}2*{log}_{10}\left({f}_{1}/{f}_{2}\right).$$

Thus, for our manipulations, we multiplied or divided the mean F0 of our speaker’s carrier sentence by the 12th root of two (i.e., 2^1/12^) four times over to calculate the upshifted and downshifted F0 (Hz) targets, respectively. We also subjected the carrier sentence to a 0-Hz mean F0 manipulation to ensure that any artifacts created during the F0 shifting process would also be present in the mid-F0 context condition. This resulted in a ~ 2-Hz difference between the original carrier sentence and the zero-shifted mid-F0 context carrier sentence. The F0 shifting was carried out using PSOLA in Praat. It is worth emphasizing that we ensured that the F0 shifting process retained both the F0 contour and the mean intensity of the original signal. The output of this procedure was three sound files with the same duration and the same intensity contour but different mean F0s. These three files constituted the three F0 contexts. Each file was 1.44 s long and the fricatives, which were the targets for splicing, were between 0.86 s and 1.05 s.

##### Merging the continuum with the F0 contexts

After the synthesis of the eight-step fricative continuum and the three F0 contexts, we used a Praat script to remove the original fricatives from the three carrier sentences and replace them with items from the synthesized continuum. We first scaled the mean intensities of each section to the values obtained from our speaker’s original recordings of the carrier sentences. That is, we took the mean intensity of “Nu komt het woord” from both the “Nu komt het woord sok” and the “Nu komt het woord sjok” recordings and averaged them to determine the target mean intensity for the pre-fricative context. Similarly, the target mean intensity for the synthesized fricatives was the average of the mean intensity of the /s/ and the /ʃ/ in the original carrier sentences. Finally, the post-fricative context /ɔk/ was shifted to the average value of the mean intensities of the sentence-final /ɔk/ from the two carrier sentences.

Further, to simulate the co-articulation that would normally occur when a Dutch speaker produces these fricatives after the voiceless stop [t], we concatenated the pre-context for each condition and each step of the synthesized continuum with a 96-ms overlap. That is, the last 96 ms of the [t] was multiplied by a falling cosine, the first 96 ms of the fricative was multiplied by a rising cosine, and the two were cross-faded over 96 ms. This amount of overlap produced a natural sounding transition from the stops to the fricatives, further concealing the artificial nature of our fricative stimuli. Finally, the 24 sound files that were outputted by our manipulation procedure were concatenated with the post-fricative context (i.e., taken from the original /ʃ/ carrier sentence) with a 2-ms overlap. This miniscule overlap was added to ensure that no intervening silences were generated by the concatenation process. The resulting 24 files are publicly available on the OSF.

#### Procedure

Upon providing informed consent, participants proceeded to a sound-attenuating booth equipped with a 24-in. monitor, stereo headphones, and a keyboard. Participants sat roughly 65 cm away from the monitor.

The experiment was programmed and executed using Presentation® software (Version 22.1, Neurobehavioral Systems, Inc., Berkeley, CA, www.neurobs.com). All participants used the same configuration of keyboard inputs, monitor settings, and, most importantly, audio settings. Presentation was configured to set the system volume level at 50%, which produced acoustic output at a comfortable listening level. Additionally, the duration of the trial event was checked and reset on every trial to ensure that the entire audio file played before the response period began.

Upon starting the experiment, participants read through the instructions which informed them about the 2AFC task and the keys they needed to press to indicate their decisions. Specifically, participants were informed that they were going to hear one of two Dutch words (*sok* or *sjok*) in a context sentence and had to indicate which one they had heard by using the keyboard. The answer keys were counterbalanced based on participant number such that participants with an even subject ID had *sok* on the left (key A) and *sjok* on the right (key L) and participants with an odd subject ID had the reverse. Participants then proceeded to practice trials which aimed to familiarize them with the testing paradigm. The practice trial conditions only included trials with the mid-F0 context and the first and eight steps of the synthesized continuum to ensure that only the smallest possible amount of conflict or ambiguity was introduced before the main task began. Thus, the practice trials consisted of six mid-F0 trials, three with /s/, and three with /ʃ/.

Once participants completed the practice trials, they proceeded to the main 2AFC task. The task had 24 trial types given the eight-step synthesized continuum and the three F0 contexts. Each stimulus was presented ten times, in ten blocks of 24 trials in random order to ensure that randomization occurred in a relatively constrained manner. That is, all 24 trial types had to be presented in a randomized fashion before the order of the next 24 was shuffled and presented. This significantly reduced the probability of participants encountering the same F0 context or the same synthesized continuum step on consecutive trials. Each trial lasted 5 s, including stimulus presentation. Thus, participants had approximately 3,700 ms to indicate their responses. After trial 80 and trial 160, participants were given self-timed breaks that they could terminate with a key press. Both at the start of the task and after each break, a 5-s countdown after the key press ensured that participants did not miss trials. During the breaks, participants were also informed about their progress explicitly (“You have now completed one-third/two-thirds of the experiment”). Our aim in communicating participant progress directly was to minimize possible loss of attention given the short duration but high repetitiveness of the task.

Upon completion of the main task, participants received a debriefing sheet explaining the aim of the experiment and signed a payment form to receive monetary compensation for their participation.

### Results

Across all ten participants and all 2,400 trials, only one trial had timed out. This was excluded from the analyses. The data are summarized in Fig. 2.

For the analysis of the categorization task, we used a generalized linear mixed-effects model with logistic linking function (Baayen et al., 2008) in package “lmerTest” (Kuznetsova et al., 2017) in R (R Core Team, 2022). The Response variable was coded as a binary variable where 1 corresponded to an/s/response and 0 corresponded to an/ʃ/response. The F0 context was contrast coded (Context Condition) such that high F0 corresponded to 0.5, mid to 0, and low to −0.5. To improve model fitting, we coded fricative continuum steps as a continuous *z*-scored variable computed by using the scale() function in R. We then generated a Quadratic Step variable by squaring the *z*-scored Scaled Step values, given that we may expect a quadratic effect of fricative step with the highest difference in response proportions appearing in the middle steps (i.e., 4–5) of the continuum. The model included random intercepts for Participants, with by-Participant random slopes for Step and Condition (Model: (response ~ (step_scaled + context * step_quad) + (1 + step_scaled + context | participant_number)).

We found that Step was the strongest predictor of response proportions (*β* = −5.01, *SE* = 0.69, *z* = −7.24, *p* < 0.001), indicating that higher steps (i.e., acoustically closer to /ʃ/) led to a lower rate of /s/ responses. Crucially, Context Condition significantly predicted /s/ response proportions (*β* = −0.90, *SE* = 0.28, *z* = −3.24, *p* = 0.001). Thus, the overall effect of F0 context on response proportions was in the expected direction. That is, in our data, low-F0 led to more /s/ responses than mid-F0, which in turn led to more /s/ responses than high-F0.

The Quadratic Step variable did not significantly predict Response (*β* = −0.04, *SE* = 0.17, *z* = −0.234, *p* = 0.81), but had a significant interaction with Context Condition (*β* = 0.75, *SE* = 0.38, *z* = 1.98, *p* = 0.047). Thus, we also found evidence for gradual reductions at the endpoints of the continuum in the differences between the different F0 conditions. This is in line with our expectation that the differences between the three F0 conditions would be the smallest in the least ambiguous steps and largest in the most ambiguous steps.

### Discussion

Overall, the results of Experiment 1 supported our hypothesis that F0 context would generate an F0-modulated contrastive bias in the perception of the fricatives where a high F0 context induced more /ʃ/ (i.e., low CoG) responses and a low F0 context was associated with more /s/ (i.e., high CoG) responses, most noticeably in the ambiguous range of the continuum. Furthermore, Experiment 1 effectively constituted a Dutch conceptual replication of the original study in German (Niebuhr, 2017) and provided another example of contrastive signal-driven effects in speech perception (Stilp, 2020).

The ceiling effects observed in Experiment 1 for the first and last few steps of the continuum demonstrated that participants did indeed perceive the first and final steps as unambiguous tokens of /s/ or /ʃ/, respectively. As expected, the validity of fricative categorization as a proxy measure for perceptual pitch adjustments was most evident in the most ambiguous steps of the fricative continuum, where additional information from the phonological context may have been necessary on account of the difficulty of categorization due to the unreliability of the spectral information in the fricatives themselves.

## Experiment 2a

Having established the contrastive effect of F0 information on fricative CoG perception in Experiment 1, we focused in Experiment 2a on the primary aim of the study, namely to test whether consistent F0 information acquired from a talker could lead to similar contrastive perceptual adjustments to information from a preceding sentence context.

The results of Experiment 1 informed the design of Experiment 2a in several ways. The validity of our artificial eight-step continuum (i.e., the response proportions displayed a consistent decline in the proportion of /s/ responses) enabled us to establish a more constrained, more ambiguous subset of the continuum. Therefore, to ameliorate the repetitiveness of the categorization task while simultaneously increasing the number of trials per fricative step, we used only steps 3–7 in Experiment 2a, in light of the response ceilings observed in the first and last two steps of the eight-step continuum. Finally, the results of Experiment 1 provided a set of effect-size estimates that were used in the power analysis for Experiment 2a.

We removed the preceding F0 context of the fricative (i.e., the carrier sentence used in Experiment 1) while maintaining a mid-F0 post-fricative lexical frame (i.e., /ɔk/ with zero-shifted F0). Two groups of participants evenly distributed into low-F0 and high-F0 exposure groups listened to 20 min of speech from a female native Dutch speaker whose voice had been F0-shifted by four semitones in accordance with the F0 condition (i.e., high or low). All participants then categorized the same isolated words (i.e., /?ɔk/) containing fricatives from the five-step fricative continuum as “sok” or “sjok.” Given the evidence for the potential of the acoustic context to take priority over a possible talker effect (Maslowski et al., 2019; Reinisch, 2016b), only the post-fricative syllable /ɔk/ was used as the immediate context.

We expected that previously acquired talker information should lead to a contrastive effect on fricative CoG perception. Thus, we expected that information acquired from a high-F0 or low-F0 talker (i.e., by two distinct groups of participants) would modulate response proportions in the same direction as the F0 context effect found in Experiment 1. Specifically, we expected the low F0 group to give a significantly higher proportion of /s/ responses than the high F0 group.

### Method

#### Power analysis

We conducted an a priori simulation-based power analysis based on the results of Experiment 1 to determine a sample size for Experiment 2a, with sufficient power to detect a talker familiarity effect with outcomes comparable to the F0 context effect in Experiment 1.

The model used to estimate this sample size was different from the model used in Experiment 1 in several ways. First, the model did not include a quadratic Step predictor but included a scaled Step predictor as a continuous variable, given that Experiment 2a only used the more ambiguous steps 3 through 6, where a much more evenly distributed categorical difference between the two groups was expected. Experiment 2a (as well as the experiments that follow it) was designed with no middle F0 condition. This design decision followed from the same principle that led to the subsetting of the continuum, namely a desire to increase the trials of primary interest (i.e., ambiguous fricative steps). Given the role of middle F0 in Experiment 1 as a control condition to test the validity of the continuum, we wanted to focus more on between-group differences we expected across low F0 and high F0. Group (i.e., talker mean F0) was contrast coded (i.e., −0.5 for one talker group and 0.5 for the other) and included as a categorical predictor. Step (i.e., fricative step) was z-scored and included in the model as a categorical predictor as well. Participant number was included as a random intercept. The model did not look for an interaction between Step and Group.

We used the “mixedpower” package (Kumle et al., 2021) in R to run a high number of simulations (i.e., 3,000 over three runs of 1,000) and obtain power estimates by repeatedly simulating data (with a between-subjects talker F0 variable) and carrying out significance tests using the model described in the previous paragraph. In addition, fixed effect sizes were conservatively estimated at 85% of their original values from Experiment 1, given the tendency of pilots to be underpowered (Albers & Lakens, 2018). The simulations showed that a sample of 32 (consisting of two equally sized groups of 16) was sufficient to consistently achieve 80% power. The power analysis script is publicly available on the OSF.

#### Participants

Thirty-three adult female native speakers of Dutch (age range = 19–31, *M*_*age*_ = 24.82, *SD*_*age*_ = 3.16 years) participated in the experiment. One participant failed to respond to eight of the ten true–false questions correctly during the exposure phase. Her data were excluded, yielding a sample of 32 participants with usable data. Participants were randomly assigned to either a high F0 group or a low F0 group (*N* = 16 each). Participants gave informed consent as approved by the Ethics Committee of the Faculty of Social Sciences of Radboud University (project code: ECSW-2019–019).

#### Materials

##### Creating the exposure material

We used ten text snippets of ~ 300 words (i.e., each roughly corresponding to 2 min of speech given our speaker’s speaking rate) as scripts for the exposure recordings (see OSM on the OSF for all texts). These texts were unrelated to each other in content but were coherent within themselves. Given our primary interest in the suprasegmental F0, we had no strict criteria for the semantic content of the texts. However, highly emotional language, political content, or possible taboo words or profanities were avoided. The texts and (F0-shifted) recordings are publicly available on the OSF.

We then recorded these snippets with the same speaker as in Experiment 1, using the same audio equipment and settings. Each snippet was recorded as a separate take to maximize the quality and homogeneity of each snippet while also affording the speaker shorter re-takes where necessary.

In line with our estimate of the speaker’s speech rate, all texts together corresponded roughly to 20 min of speech. Each snippet was, on average, 121.41 s long with a standard deviation of 10.61 s. The cumulative duration of all audio files used for the exposure task was 20 min and 24 s, including ~ 2 s of leading and trailing silences for each file. The 2-min snippets were then F0-shifted by + 4 and ˗4 semitones to create the high and low F0 conditions of the experiment, respectively. The speaker’s mean f0 across the ten recordings was 223 Hz. Thus, the mean f0 in the low F0-shifted files was ~ 185 Hz, and the mean f0 in the high F0-shifted files was ~ 293 Hz. The calculation of the precise target values in Hertz and the shifting were done using the same Praat script as was used in Experiment 1.

In addition to the auditory stimuli, to assess participants’ listening performance, we prepared ten true–false content questions, one for each 2-min snippet (see OSM on the OSF for all questions and responses). The questions all began with phrases such as “*Volgens dit rapport…”* (“According to this report…”) or “*Volgens de tekst”* (“According to the text”), to ensure that participants’ responses exclusively relied on information that was presented in the task. The correct responses to the true–false questions were half true and half false.

##### Creating the word conditions for the categorization task

To adapt the stimuli from Experiment 1 to the word level, we extracted the words “/?/ok” from steps 3–7 of the mid-F0 condition in Experiment 1. Given that the final stimuli in Experiment 1 simulated coarticulation at the *“…woord sok/sjok”* word boundary, the onset of the word-level stimuli for Experiment 2a was based on the manual selection of the earliest time point of the artificial fricative following the burst of the plosive [t] (see OSM on the OSF for stimuli). Since all stimuli for Experiment 1 were generated using the same process, the differences between these time points were measured in tenths of milliseconds on account of miniscule differences between the locations of the nearest zero crossings for each boundary. The offsets for the target words were identical to those of the sentences used in Experiment 1. Thus, the five words used in Experiment 2a were identical to each other in duration down to the millisecond, and otherwise identical to the final words of the steps 3–7 mid-F0 context sentences used in Experiment 1.

##### Post-test questionnaire

Following the experiment, we presented a short questionnaire to test whether the participants had noted our acoustic manipulations or other design-related or technical issues. No specific analysis or exclusion criteria were defined for responses to the questionnaire before the experiment was carried out, as the questionnaire was primarily intended to help track the impact of the methodological changes between experiments. This list of questions used in the questionnaire began with a general feedback question and gradually became more specific with respect to the acoustic manipulations (see OSM on the OSF for questions and answers).

#### Procedure

Participants first received an information sheet about the experiment, and after giving informed consent, proceeded to the same sound-attenuating booth as in Experiment 1.

It is worth noting that participants never encountered the expressions “exposure task” or “2AFC task” at any point before or during the experiment. Instead, to help conceal the aim of the experiment, the two tasks were simply referred to as “*de luistertaak”* (“the listening task”) and “*de categorisatietaak”* (“the categorization task”). For the sake of clarity, however, this article always refers to the tasks as “exposure task” and “categorization task.”

Before the exposure task, participants saw a generic introductory screen informing them that they had to perform two different tasks, a listening task and a categorization task, and that the whole experiment was expected to take about 45 min at most. Then, instructions for the exposure task indicated that participants had to listen to ten 2-min snippets and answer a true–false question after each snippet, pertaining to the content of the snippet. Participants were also informed that the answer key (i.e., the letters “A” and “L”) and the words “*waar”* (“true”) and “*onwaar”* (“false”) would be visible when a question appeared. A final set of instructions remarked that the expectation was that participants would be able to answer at least eight out of ten questions correctly.

After reading through the instructions, participants proceeded to the exposure task. During playback, only the caption “*Luister!”* (“Listen!”) was visible on screen. After playback finished, the true–false question about the snippet appeared. The true–false question trials did not have a timeout limit. Thus, participants had to respond to each question. They received feedback for their responses. The order of the snippets and the answer key was fixed across participants. After listening to and answering the dedicated true–false question for all ten 2-min snippets, participants were informed that they had reached the end of the exposure task.

Then, in the categorization task, participants were instructed to indicate which word they had heard out of two alternatives. Although participants heard five different trial types throughout the task (i.e., given the five fricative steps), the instructions emphasized that they would “always hear one of two words, *sok* or *sjok.*” During each trial, the auditory stimulus played and the response options only appeared when playback finished. The auditory stimuli were grouped in mini-blocks of five to ensure sufficient trial-type variation in consecutive trials. The on-screen answer key (i.e., the location of the words *sok* and *sjok* and letters *A* and *L*) was randomized and evenly distributed across all participants (fixed per participant) and was on-screen throughout the response periods. Participants had 3 s (after playback) to respond to each trial and the trials were presented with a 500-ms interval in-between. The task consisted of 160 trials, with self-paced breaks every 40 trials.

After finishing the categorization task, participants proceeded to the post-test questionnaire. Participants gave self-paced typed answers to the five feedback questions. To prevent participants from guessing what a *correct* response to these feedback questions might be, participants only saw one question at a time and were not allowed to return to a question they had already answered. They were also informed that responses to the feedback questions were optional.

Upon completing the post-test questionnaire, participants received a debriefing sheet and a payment form.

### Results

**Fig. 3:**
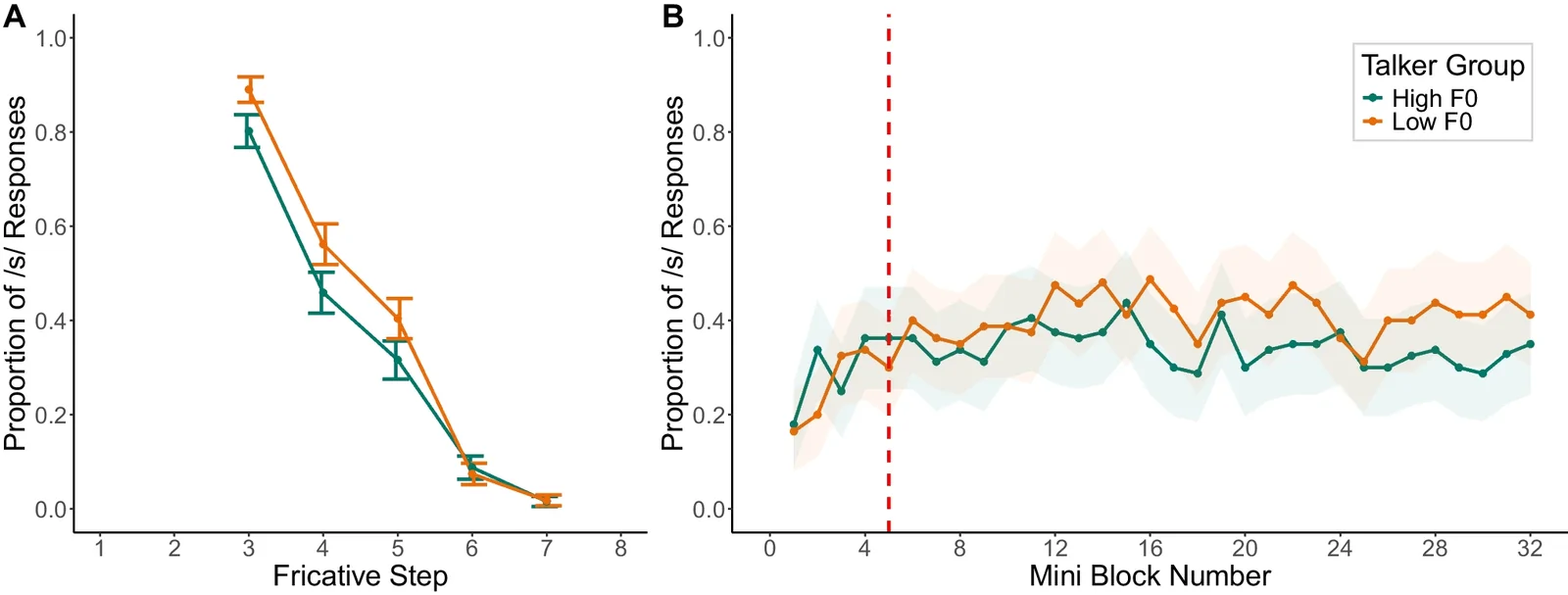
Response proportions across talker groups and mini-blocks in Experiment 2a. Figure 3 A shows the proportions of /s/ responses by fricative step and talker (F0) group, and Fig. 3B shows the proportions of /s/ responses across mini-blocks of five trials for the two talker groups. Talker groups are indicated by line color (green = high F0, orange = low F0). In Fig. 3 A, the error bars and ribbons indicate 95% confidence intervals. Figure 3 A shows that participants’ responses were once again predicted by the fricative continuum’s steps, as traversing through the/s/-/ʃ/(i.e., high CoG-low CoG) continuum consistently results in lower proportion of/s/responses. Differences in /s/ response proportions between the two talker groups are visible in Fig. 3 A, following an early bias for /ʃ/ exhibited by both talker groups. Figure 3B clearly displays this bias in the early trials of the experiment. The dashed red line on Fig. 3B marks the point (the 26th trial) after which the effect of talker group on/s/response proportions becomes significant. The divergence between the two talker groups’ response proportions grows in the latter half of the experiment. Note that Fig. 3 A lacks data points for fricative steps 1, 2, and 8 as these were not used in Experiment 2 on account of response ceilings (for steps 1 and 2) and floors (for step 8) observed in Experiment 1 (see Fig. 2A). Similarly, there was no mid F0 condition as Experiment 2’s between-subjects design did not vary test F0 and a between-group difference could be detected using only two talker groups

A total of 5,112 trials were used in the analyses of Experiment 2a after the exclusion of eight timeouts. The results are summarized in Fig. 3.

For the analysis of the categorization task, we used a generalized linear mixed-effects model with logistic linking function (Baayen et al., 2008) in package “lmerTest” (Kuznetsova et al., 2017) in R (R Core Team, 2022). The Response variable was coded as a binary variable where 1 corresponded to an/s/response and 0 corresponded to an/ʃ/response. Talker F0 Group was contrast coded (Group; −0.5 for low F0, 0.5 for high F0). The Step variable was z-scored. Next to random intercepts for Participants, the model also included by-Participant random slopes for Group and Step (Model: (response ~ (step_scaled + talker) + (1 + step_scaled + talker|ppid)).

In this initial model, Step strongly predicted response proportions (*β* = −2.30, *SE* = 0.13, *z* = −17.86, *p* < 0.001), further affirming the reliability of our continuum. As predicted, higher talker F0 was numerically associated with lower /s/ response proportions, but the difference between the two talker F0 groups was not significant (*β* = −0.74, *SE* = 0.43, *z* = −1.74, *p* = 0.081). In addition, we observed an overall bias for /ʃ/ responses throughout the experiment, with 64% of all responses being /ʃ/.

A second model was run with the factor Miniblock (i.e., a group of trials consisting of all five steps, randomly shuffled; z-scored) included in the fixed-effect structure with an interaction term between Miniblock and Group. This model revealed a significant effect of Miniblock (*β* = 0.18, *SE* = 0.05, *z* = 4.43, *p* < 0.001) as well as an interaction between Group and Miniblock (*β* = −0.33, *SE* = 0.08, *z* = −4.02, *p* < 0.001). These two outcomes suggest that participants’ responses were influenced by the experimental procedure (i.e., trial/miniblock number) and that this effect significantly changed over the experiment (see Fig. 3B). The main effect of Group, which had not reached significance in the first model, was unchanged.

Based on the results of the second model, we suspected that an early /ʃ/ bias in both talker groups might have masked the hypothesized talker effect. Visualizations of our data were also pointing at a large /ʃ/ bias which gradually disappeared over time (see Fig. 3B), and, more crucially, at the emergence of the predicted talker effect in later trials. To determine where exactly in the experiment this shift occurred, we conducted a separate set of analyses. We analyzed 60-trial subsets of the entire dataset, starting from the first trial. Thus, trial periods 1–61, 5–66, etc. were analyzed using a model which included Step and Mini-Block as fixed predictors (both scaled), and next to random intercepts by Participants, also a random slope of Mini-Block by Participant (Model: (resp ~ (step_scaled + miniblock_scaled) + (1 + miniblock_scaled|ppid)). The results revealed that 26–86 was the first trial interval without a significant effect of mini-block. Thus, we concluded that analyses from the 26th trial onward (~ 84% of the complete dataset) would be more insightful with respect to a possible effect of talker F0 without the /ʃ/ bias. It is worth noting that analyses which exclude a higher number of early trials provided larger effect sizes, and that the 26th trial cutoff was simply the *bare minimum* required to eliminate the mini-block effect.

Based on this insight, we re-analyzed a subset of the full dataset which only contained data from the 26th trial onwards (4,315 trials). The structure of this model was identical to the model used in the first round of analyses. In this model, Step was once again the strongest predictor of Response (*β* = −2.58, *SE* = 0.16, *z* = −16.49, *p* < 0.001). Critically, the model also revealed listeners in the low F0 condition had a significantly larger proportion of/s/responses (*β* = 0.91, *SE* = 0.46, *z* = 1.98, *p* = 0.0477). Thus, while responses strongly aligned with the spectral characteristics of the fricative continuum, the results of this exploratory analysis suggested that listeners’ fricative perception displayed a contrastive bias driven by previously acquired talker information.

### Discussion

Analyses of Experiment 2a involving the entire dataset pointed at a strong /ʃ/ bias in both talker F0 groups in the early stages of the experiment (i.e., the first 25 trials). Subsequent analyses revealed a significant effect of talker F0 when data from the first 25 trials were excluded. We attribute this early /ʃ/ bias to participants’ learning about the fricative endpoints and the acoustic space covered by the continuum. This learning/training process may have been necessary for participants due to the reduced reliability (i.e., distance from existing Dutch fricative perceptual categories) of the five-step subset of the original eight-step continuum. In particular, we speculate that the lack of a strong /s/ endpoint may have been a major factor, as underlined by the absence of an /s/ response proportion ceiling on Step 3, which was present in the categorization task in Experiment 1. Thus, participants may have only made use of talker information after adjusting to the relatively unreliable spectral features of the fricative continuum.

The mini-block effect and the consequent early /ʃ/ bias observed in Experiment 2a prompted the design of multiple follow-ups, including Experiment 2b reported below. Two additional follow-up experiments have already been reported in (Uluşahin et al., 2024), using a different five-step subset of the original eight-step continuum to the one used in Experiments 2a (i.e., steps 2–6 as opposed to steps 3–7) and implementing practice trials with the original endpoints (i.e., steps 1 and 8). These changes successfully eliminated the early bias, but the data revealed a talker F0 effect that was not in the expected contrastive direction, whereby hearing high F0 led to higher CoG perception (i.e., more /s/ responses). We attributed this effect not to a direct assimilatory mechanism, but to an interaction of two contrastive mechanisms. Namely, we speculated that familiarization with the talker’s F0 led to a contrastive adjustment in the perception of the post-fricative vowel (i.e., /ɔ/), which in turn had a backwards contrastive effect on the perception of the preceding target fricative. Further research will be required to investigate the cause of this non-contrastive effect. The goal of Experiment 2b, however, was simply to establish if there are conditions under which there is a reliable contrastive talker-bound F0 effect on fricative CoG perception.

## Experiment 2b

Given the unexpected mini-block effect and the delayed talker F0 effect observed in Experiment 2a, we ran a follow-up experiment which implemented one major design change: Experiment 2b used the entire eight-step fricative continuum rather than the five-step subset used in Experiment 2a. This change aimed to minimize the impact of a potential early /ʃ/ bias by allowing participants to become familiar with the continuum more quickly.

### Method

#### Participants

Recruitment criteria in Experiment 2b were identical to those of Experiment 2a. Thus, another group of 34 healthy adult female native speakers of Dutch (age range = 18–32, *M*_*age*_ = 23, *SD*_*age*_ = 3.51 years) participated. Two of the first 32 participants failed to respond to 80% of the true–false questions in the exposure task. Their data were excluded, and two others were tested in their place to achieve a sample size of 32. Participants were randomly assigned to either a high F0 group or a low F0 group (*N* = 16 each). Participants gave informed consent as approved by the Ethics Committee of the Faculty of Social Sciences of Radboud University (project code: ECSW-2019–019).

#### Materials

The materials used in the exposure task were identical to those in Experiment 2a. For the categorization task, the remaining three steps of the continuum were added to the stimuli to restore the original continuum.

#### Procedure

The experimental procedure was identical to that of Experiment 2a with one notable exception: The number of trials in the categorization task was increased from 160 to 240 in order to accommodate the higher number of fricative continuum steps.

### Results

**Fig. 4:**
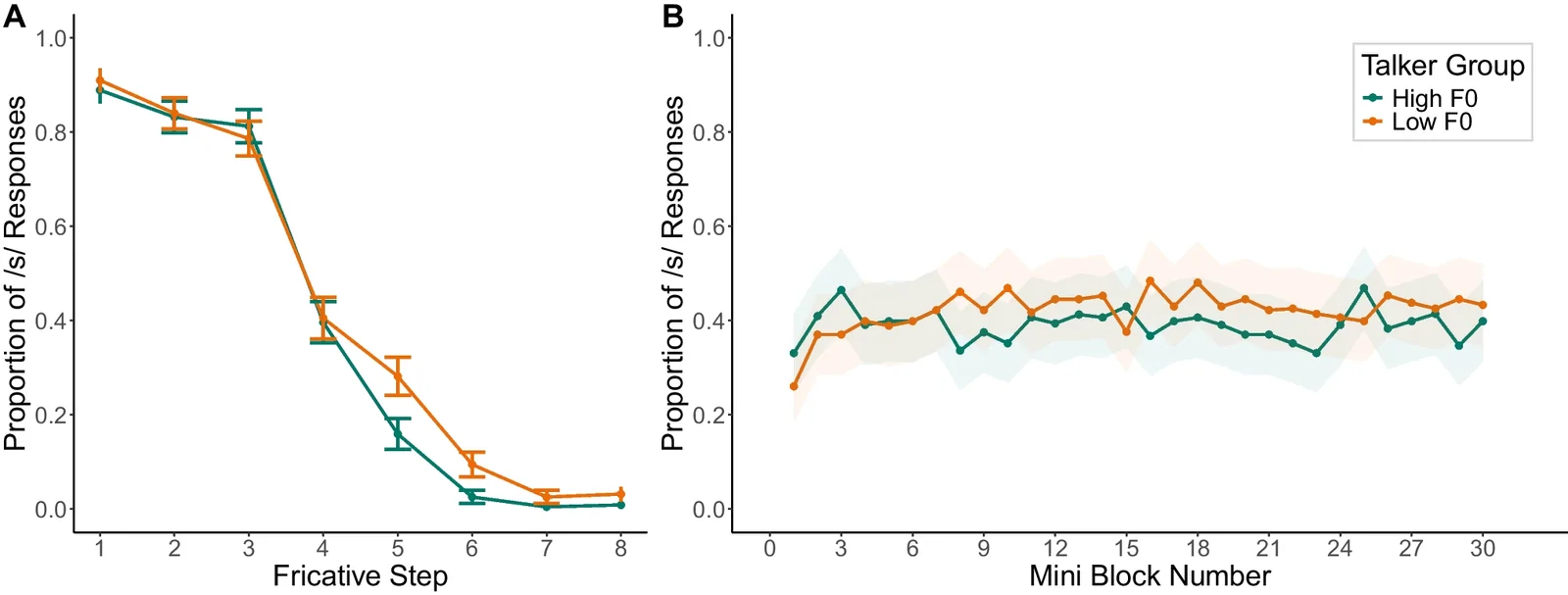
Response proportions across talker groups and mini-blocks in Experiment 2b. Figure 4 A shows the proportions of /s/ responses by fricative step and talker (F0) group, and Fig. 4B shows the proportions of/s/responses across mini-blocks of eight trials for the two talker groups. Talker groups are indicated by line color (green = high F0, orange = low F0). Experiment 2b was a replication of Experiment 2a with one crucial change: The restoration of the full eight-step fricative synthetic continuum at test. The exposure phase, and thus the talker F0 information presented to participants, were identical. In Fig. 4 A, the error bars and ribbons indicate 95% confidence intervals. Figure 4 A shows a consistent decline in/s/response proportions over the eight-step fricative continuum, indicating that participants’ responses were guided by the fricative continuum. Crucially, the figure also displays a difference in/s/response proportions between the talker groups in the middle steps of the continuum. A contrastive effect seems to guide participant responses such that participants in the high F0 talker group hear the same continuum as being more /ʃ/-like (i.e., low CoG), and participants in the low F0 talker group hear it as being more/s/-like (i.e., high CoG). Additionally, Fig. 4B displays a shorter and smaller early/ʃ/bias in Experiment 2b compared to Experiment 2a (see Fig. 3B), statistically corroborated by the absence of a main effect of mini-block in analyses. Consequently, the talker effect visible on Fig. 4 A is reliably detectable across the experiment

With 32 timed-out trials, all analyses of the categorization task were based on a total of 7,648 trials. The results of Experiment 2b are summarized in Fig. 4.

For the categorization task of Experiment 2b, we conducted a preliminary analysis which included Step (z-scored), Group (contrast coded; low F0 = −0.5, high F0 = 0.5), and Miniblock (z-scored) as fixed effects with all interaction terms, random intercepts for Participants, and by-participant random slopes for all fixed effects. Within this model, Step was once again the strongest predictor of/s/response proportions (*β* = −3.25, *SE* = 0.25, *z* = −12.96, *p* <.001). Crucially, we found an effect of Group (*β* = −0.62, *SE* = 0.27, *z* = −2.32, *p* =.02), indicating that participants were using the F0 information they had acquired in the exposure task to later categorize incoming fricatives in the 2AFC task. Additionally, we found no effect of Miniblock (*β* = 0.15, *SE* = 0.11, *z* = 1.38, *p* =.17), although it significantly interacted with Step (*β* = 0.15, *SE* = 0.06, *z* = 2.57, *p* = 0.01), suggesting that later trials in the experiment elicited a slightly higher proportion of /s/ responses. Finally, we observed a three-way interaction between all three terms (*β* = −0.52, *SE* = 0.12, *z* = −4.91, *p* <.001), suggesting that, in addition to a steady decline in /s/ response proportions over increasing fricative steps, participants in the high F0 talker group gave an even lower proportion of /s/ responses as the experiment progressed.

Given the absence of a Miniblock effect over all trials in the preliminary model, we re-analyzed the dataset with only Step (z-scored) and Group (contrast coded; low F0 = −0.5; high F0 = 0.5) to obtain a better estimate of the talker effect size. Once again, the model included an interaction term as well as random intercepts for Participants and by-Participant random slopes for Step and Group. This main model revealed a significant effect of Step (*β* = −3.09, *SE* = 0.26, *z* = −11.99, *p* <.001), Group (*β* = −0.70, *SE* = 0.26, *z* = −2.67, *p* =.008), and a significant interaction between the two (*β* = −1.22, *SE* = 0.51, *z* = −2.36, *p* =.02). Here, critically, the effect of Group indicates that participants used F0 information acquired in the exposure task to disambiguate incoming fricatives. The large effect of Step indicates that the continuum functioned as intended. Finally, the interaction indicates that the size of the Talker F0 effect significantly increased across the continuum steps.

### Discussion

The extension of Experiment 2a’s design to include the entire 8-step fricative continuum in Experiment 2b successfully eliminated the Miniblock effect. Consequently, we observed a consistent Talker F0 Effect that persisted throughout the experiment, despite the presence of a shorter and smaller (i.e., compared to Experiment 2a) early /ʃ/ bias. This result suggests that listeners are indeed able to use earlier acquired talker F0 information to disambiguate fricatives. However, given the miniscule amount of F0 information embedded in the immediate acoustic context (i.e., a fixed-F0 /-ɔk/), Experiment 2b (like Experiment 2a) cannot speak to whether or how talker F0 information may be utilized for the same purpose when there is also sufficient local contextual F0 information. Experiment 3 investigated this question.

## Experiment 3

This online experiment aimed to put consistent and reliable low-variation talker F0 information acquired over 20 min of listening and high-variation contextual F0 information (i.e., multiple F0 contexts at test) in direct competition. Specifically, one group heard a high F0 talker in exposure while the other heard a low F0 talker, but both groups performed the same test phase (i.e., 2AFC task) with an even mix of high and low F0 carrier sentences. This design could reveal one of several outcomes. In the event that we find a context F0 effect but no talker F0 effect, the data would provide evidence in favor of theories which assert that access to talker information may be reweighed (e.g., Martin, 2016) based on the ability of contextual information to successfully disambiguate incoming speech (i.e., similar to how local speech rate "takes over" the tracking of habitual speech rate; e.g., Maslowski et al., 2019; Reinisch, 2016b). Within this paradigm, we hypothesized that both (i) contextual and (ii) talker cues would lead to contrastive effects on fricative perception. Additionally, (iii) we expected the continuum to lead to an increasingly /ʃ/-like perception over steps, and (iv) we expected potential context and talker effects to both be largest in the more ambiguous steps of the continuum.

### Method

#### Participants

Participant recruitment for Experiment 3 was subject to the same constraints as the previous experiments. Thirty-two healthy adult female native speakers of Dutch (age range = 21–41, *M*_*age*_ = 26.97, *SD*_*age*_ = 5.15 years) participated in the experiment. Participants were recruited online through Prolific. Participants were randomly assigned to one of two even (*N* = 16) groups: A high F0 exposure-low group or a low F0 exposure group. Participants gave informed consent as approved by the Ethics Committee of the Faculty of Social Sciences of Radboud University (project code: ECSW-2019–019).

#### Materials

Experiment 3 combined materials from both prior experiments, using the full eight-step continuum with carrier sentences from Experiment 1 as well as the exposure recordings from Experiment 2a.

In addition, a headphone check was implemented into the online testing environment to assess whether participants were wearing stereo headphones during the experiment. This headphone check utilized an online adaptation of the Huggins pitch phenomenon whereby pitch perception in white noise is only possible through binaural signal segregation in stereo headphones (Cramer & Huggins, 1958; Milne et al., 2021).

#### Procedure

Participants who followed the Prolific link to the experiment were forwarded to a consent form. After providing informed consent, participants proceeded to the headphone check. The headphone check involved 12 Huggins pitch trials (Cramer & Huggins, 1958) in which participants had to pick one of three signals as containing a faint tone embedded in noise, with the other two being white noise fillers (see Milne et al., 2021, for task details). Participants who failed to respond to eight out of 12 trials correctly in the headphone check were informed that they could not participate and were redirected to the recruitment platform. Participants who passed the headphone check proceeded to the listening task. The listening task was identical to that of Experiments 2a and 2b.

After the listening task, participants proceeded to the categorization task. The categorization task in Experiment 3 was identical to the same task from Experiment 1 with one major change: The mid-F0 context condition was removed. Thus, 240 trials were equally distributed across eight fricative steps and two F0 conditions, creating 15 mini-blocks consisting of 16 trials each.

After the categorization task, participants completed a post-experiment questionnaire, which was the same as in Experiment 2a, but with two new questions: One asking whether participants thought there were two different talkers in the categorization task and one asking which particular pair of headphones/earbuds they were using (see OSM on the OSF for a full list of questions).

### Results

**Fig. 5:**
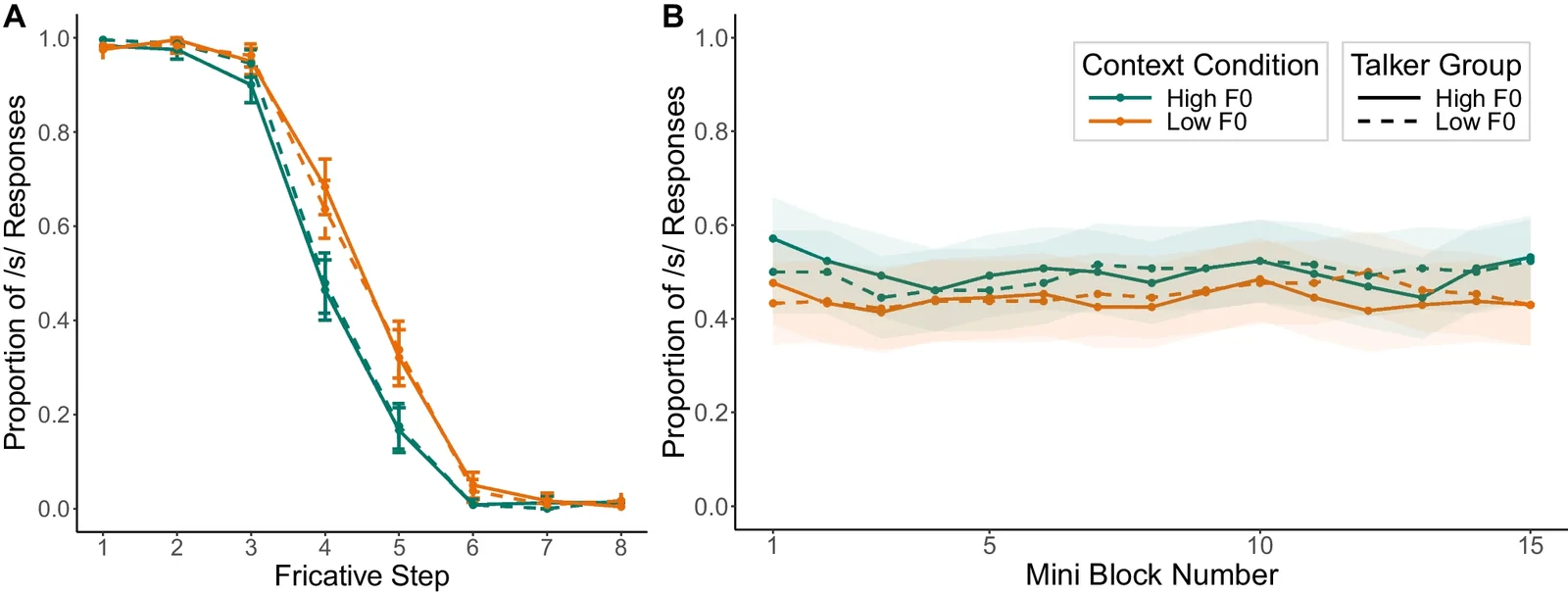
Response proportions across talker groups, context conditions, fricative steps, and mini-blocks in Experiment 3. Figure 5 A shows the proportions of /s/ (i.e., high CoG; as opposed to low CoG /ʃ/) responses by fricative step, context (F0) condition, and talker (F0) group, and Fig. 5B shows the proportions of /s/ responses across mini-blocks of 16 trials for the two talker groups and context S conditions. Talker groups are indicated by line type (solid = high F0 talker group, dashed = low F0 talker group), and context condition at test is indicated by line color (green = high F0, orange = low F0). In Fig. 5 A, the error bars and ribbons indicate 95% confidence intervals. Figure 5 A displays that participants’ responses were guided primarily by continuum step, similar to previous experiments. In addition, the contrastive context condition effect originally found in Experiment 1 is replicated with a clear divergence in response proportions between the two context conditions. Crucially, this is accompanied by the absence of a talker group effect observed in Experiment 2b, suggesting that, when sufficient F0 information is present in the acoustic context, listeners might not rely on talker F0 information. Figure 5B displays the lack of significant differences within and between the context condition and talker group manipulations over the course the experiment. In addition, both the global and the early /ʃ/ biases are absent. Thus, the results of Experiment 3 strongly resemble those of Experiment 1, with a clear context condition effect and no evidence for a talker group effect

All participants responded to the true–false questions in the exposure task with at least 80% accuracy. Thus, no participants were excluded and there were no re-tests. The results of Experiment 3 are summarized in Fig. 5.

For the analysis of the categorization task, we used a generalized linear mixed-effects model with logistic linking function (Baayen et al., 2008) in package “lmerTest” (Kuznetsova et al., 2017) in R (R Core Team, 2022). A total of 7,668 trials were used in the analyses after the exclusion of 12 timeouts. The Response variable was coded as a binary variable where 1 corresponded to an/s/response and 0 corresponded to an /ʃ/ response. Talker F0 Group (Group) and F0 context (Context Condition) were both contrast-coded with low F0 corresponding to −0.5 and high F0 to 0.5. The model also included an interaction between Group and Context Condition. The Step variable was z-scored. The model also included random intercepts for Participants and by-participant random slopes for Step and Group. A random slope for Context Condition was excluded as observed variation was near-zero, and models including the random slope produced singular fits.

Within this model, Step strongly predicted response proportions (*β* = −5.50, *SE* = 0.41, *z* = −13.51, *p* < 0.001). Context Condition also significantly predicted response proportions (*β* = −0.87, *SE* = 0.10, *z* = −9.10, *p* < 0.001) while Group did not (*β* = −0.05, *SE* = 0.40, *z* = −0.13, *p* = 0.90). Furthermore, we found no interaction between Context Condition and Group (*β* = −0.16, *SE* = 0.19, *z* = −0.85, *p* = 0.39). The strong effect of Step once again indicated that the fricative continuum functioned as intended. The Context Condition effect replicated the findings of Experiment 1 with comparable effect sizes, suggesting that the contrastive effect of Context Condition on the perception of fricative CoG persists even when additional F0 information is provided through talker exposure. The lack of a Group effect indicates that there was no evidence that participants’ responses were affected by the F0 information they acquired during the 20-min exposure task.

We also conducted a round of checks for a possible mini-block effect in Experiment 3. The model structure was identical to the one used in Experiment 2a to specify the boundaries of the mini-block effect (Model: (resp ~ (step_scaled + miniblock_scaled) + (1 + miniblock_scaled|ppid)). However, this time, the model was run with the entire dataset instead of subsets of 60 trials. Within this model, Step once again significantly predicted /s/ response proportions (*β* = −2.39, *SE* = 0.17, *z* = −13.94, *p* < 0.001) whereas Mini-block did not (*β* = 0.04, *SE* = 0.10, *z* = 0.47, *p* = 0.64). Thus, participants’ response proportions over the experiment were not modulated by an order bias.

Finally, to test the ambiguity hypothesis, we computed a Quadratic Step variable by squaring the (scaled) Step variable, and ran a model which included (scaled) Step, F0 Context, and Quadratic Step as fixed predictors with an interaction between F0 Context and Quadratic Step, and random slopes for (scaled) Step by Participant. We excluded random slopes for F0 Context from the model as its variance was too small to be supported by the dataset and the model produced singular fits. Based on this analysis, we found that Step (*β* = −5.52, *SE* = 0.41, *z* = −13.33, *p* < 0.001) and F0 Context (*β* = −1.11, *SE* = 0.11, *z* = −9.90, *p* < 0.001) significantly predicted response proportions. The Quadratic Step variable did not have a main effect (*β* = −0.12, *SE* = 0.13, *z* = −0.91, *p* = 0.36), but displayed a significant interaction with the F0 Context (*β* = 0.97, *SE* = 0.23, *z* = 4.27, *p* < 0.001). This suggests that, as predicted, the effect of the local F0 context was heavily modulated by fricative ambiguity whereby the continuum endpoints (i.e., steps 1 and 8) displayed the smallest context effects, and the middle steps of the continuum (i.e., steps 4 and 5) displayed the largest (see Fig. 5A).

#### Bayes factor analysis

The design of Experiment 3 could theoretically detect a context effect and a talker effect simultaneously. However, based on the data from Experiments 1, 2a, and 2b, one could expect the talker effect to be smaller and more variable than the context effect. Therefore, it is possible that a talker effect was present, but simply too small to be detected by our analyses. To address this issue, we ran Bayes factor (BF) analyses. These analyses used the model estimates from Experiment 1 and Experiment 2b as priors for the context and talker effects, respectively. For the talker effect, we obtained a BF of 0.31, indicating that the null hypothesis was more than three times as likely as the alternative hypothesis. For the context effect, we obtained a BF of 3.03 (see OSM on the OSF for analysis scripts and results). Thus, the former analysis provided moderate evidence for the null (i.e., no talker effect), and the latter provided moderate evidence (Kruschke, 2021; Lee & Wagenmakers, 2014) for the alternative (i.e., context effect), which had already been detected by the frequentist analyses.

### Discussion

The design of Experiment 3 combined the context F0 manipulation of Experiment 1 and the talker F0 groups of Experiments 2a and 2b. Therefore, participants could use both contextual and talker-bound F0 cues to disambiguate tokens from the fricative continuum. While the context effect observed in Experiment 1 was replicated (with a highly similar effect size and variability), we found no evidence for the talker effect observed in Experiment 2b and the later trials of Experiment 2a. Thus, talker information may not be accessed or actively used when the acoustic signal carries enough contextual information for the successful disambiguation of the fricatives.

## General discussion

The human brain constantly utilizes a wide array of strategies to address the infinite variability of speech. A considerable part of this process depends on cues present in the incoming acoustic signal. Thus, it is crucial for researchers to determine which cues are used when, and which perceptual outcomes different strategies lead to. To this end, the present study investigated the role of contextual and talker F0 information on fricative perception. Our primary aim was to test whether talker-specific F0 information could lead to perceptual adjustments in the same direction as context effects (i.e., usually contrastive, but not always; see Stilp, 2020), and, if a talker effect was found, to investigate how it compared to the context effect. We had two primary hypotheses: We predicted that participants’ response proportions to a synthesized /s/-/ʃ/ continuum would show a contrastive effect of F0 information, both (i) present in the acoustic context (i.e., F0 context effect) and (ii) available as talker information (i.e., F0 talker effect).

In Experiment 1, F0 context elicited responses that were in line with the hypothesized contrastive relationship between mean sentence F0 and target fricative CoG in perception, where low sentence F0 led to more /s/ (i.e., high CoG) responses and high sentence F0 led to more /ʃ/ (i.e., low CoG) responses. These findings further validated fricative CoG perception as a proxy measure of perceptual adjustments to F0. They also validated the experiment’s role as a stepping stone to Experiments 2a and 2b, which relied on the same proxy measure, but instead tested effects of F0 information coming from the talker rather than the acoustic context.

Experiment 2a investigated whether contrastive effects of talker-bound F0 information could be observed within a similar paradigm. Two participant groups first listened to the same talker reading out stories at two different average F0s, after which they categorized a single-word /s/-/ʃ/ continuum (i.e., without any preceding sentential context). The two groups (i.e., high F0 and low F0) only diverged in their fricative categorization responses in the direction consistent with the hypothesized talker effect after an initial bias for /ʃ/ responses. That is, participants who heard a low F0 talker during exposure gave more /s/ responses and participants who heard a high F0 talker during exposure gave more /ʃ/ responses, but this was only statistically reliable after the first 25 trials (i.e., after 16% of the categorization task, by which point the early /ʃ/ bias had weakened). Experiment 2b replicated the design of Experiment 2a, but with the entire eight-step continuum to help participants become familiar with the continuum quicker, and eliminate the early /ʃ/ bias observed in Experiment 2a. This was successful as we saw a smaller and shorter bias which was also not detected as a main effect of miniblock in the analyses. Critically, we found a robust contrastive effect of talker F0 on response proportions throughout the experiment, indicating that participants were actively using talker F0 information during the normalization of the ambiguous fricatives, and that fricative CoG perception was a valid way to measure adjustments to talker F0 information as well as contextual F0 information.

In Experiment 3, we combined the context F0 manipulation of Experiment 1 with the talker F0 manipulation of Experiments 2a and 2b. Participants were not only divided into high and low F0 talker groups, but they all also heard high and low F0 carrier sentences before the target fricatives in the categorization task. Thus, participants were provided with adequate F0 information to disambiguate the target phonemes from both the acoustic context and talker information. Here, we found a large effect of F0 context, which was comparable in size to the analogous effect observed in Experiment 1. Crucially, our analyses did not reveal a talker effect, as there were negligible differences in response proportions across the two talker groups. A Bayesian factor analysis (BFA), which found moderate evidence in favor of the null (i.e., no talker effect), corroborated this result.

The context effects in Experiments 1 and 3 highlight how listeners can use F0 information in the context to disambiguate fricative CoGs, and how access to contextual information may only be necessary when the target phoneme is unreliable. Crucially, the talker effect found in the fixed-F0 categorization task used in Experiments 2a and 2b sharply contrasts with the lack of a talker effect in Experiment 3, which contained varying contextual information in the categorization task while using the same exposure materials as in Experiments 2a and 2b. Perhaps most surprisingly, despite using a stronger indicator of talker identity than speech rate (i.e., F0), we found that our participants’ perception was subject to the same constraints that were reported for the contrastive effects of speech rate on the perception of vowel duration and VOT (e.g., Maslowski et al., 2019; Reinisch, 2016b; Ting & Kang, 2023). That is, when contextual information was sufficient for the disambiguation of incoming speech, we found no evidence for the active use of talker information, suggesting that the successful disambiguation of the incoming signal through contextual cues either prevented or significantly outweighed the relative contribution of knowledge-driven processes dependent on talker cues, potentially compounded by increased variation at test in Experiment 3. This reweighting of talker information by contextual information is consistent with a model of speech perception involving temporally distinct loci for different types of perceptual processes (e.g., Reinisch & Sjerps, 2013; Sjerps & Reinisch, 2015). For instance, our results support the position that acoustic context effects precede most other processing (e.g., those modulated by higher-level representations like talker representations) in the perceptual stream, occurring at a lower level of processing (i.e., early locus) than talker effects or other effects that might be modulated by higher order representations (Assgari & Stilp, 2015; Bosker et al., 2017). Alternatively, within a cue-integration framework (Martin, 2016), one can view these findings as evidence for the reweighting of talker cues by the relative evidence (i.e., the confidence of the signal-driven mechanism) of contextual cues. Specifically, if the signal-driven process of context-based normalization successfully disambiguates an incoming target phoneme using only information embedded in the acoustic context, the perception system might reduce the weight of other cues, even though the process for talker normalization may still take place.

This outweighing of talker information by contextual information calls into question the relative utility of talker information in natural spoken communication. If a “good enough” signal is always going to outweigh the listener’s information about the talker, why do listeners store information about talkers for long periods of time? One answer to this question may be that knowledge about how talkers speak is necessary for talker identification. Although any talker-specific aspect of speech processing may be subordinate to acoustic cues in incoming speech, successful identification of talkers depends on the tracking and correct matching of talker-specific features. Another answer concerns the role of talker information in adverse listening conditions. Although our participants were tested in sound-attenuating booths, real-life conversations often take place alongside speech and nonspeech noise, which are likely to pose intelligibility challenges. The resulting reduction in the reliability of contextual cues may result in an increased reliance on talker information. Thus, listeners may benefit from storing talker information beyond what is strictly necessary for talker recognition in everyday settings. Indeed, this is in line with previous research reporting a ceiling for voice recognition after 10 min of familiarity, but progressively higher intelligibility benefits for exposure up to (at least) an hour (E. Holmes et al., 2021).

It is worth noting that, in order to minimize acoustic variation beyond our dependent variable of F0, our experiments only used one (F0-shifted) talker, and not multiple talkers. Consequently, our ability to speak to the talker-specificity of participants’ tracking of talker F0 information in Experiments 2a and 2b is limited. Future studies may therefore assess whether the observed F0 talker effects are indeed talker-specific by pairing different multi-talker training conditions with the same test condition. We also consider that our present methodology could not reliably determine whether participants in Experiments 1 and 3 thought there were multiple talkers with multiple F0 profiles in the categorization task, or whether they thought there was one talker at different parts of her F0 range. The perception of multiple talkers could introduce a risk of interference by unaccounted sociolinguistic variables. The post-experiment questionnaire of Experiment 3 included a question about this (see OSM for responses), and 65% of participants (i.e., 21/32) reported perceiving two different talkers. However, the limitations mentioned in this paragraph may not have significant bearing on perceptual adaptations as there is evidence for talker-specific adaptation irrespective of a listener’s explicit recognition of a voice as familiar (E. Holmes et al., 2018). Thus, these data must be interpreted carefully, as participants’ perception during the experiment or their insight into their perception of the talker in hindsight might be sub-par indicators of this aspect of the normalization process. If future work aims to investigate talker-specificity at a finer level, it might be particularly beneficial to implement a design that actively cues or provides feedback about talker identity (e.g., a written prompt or a picture clearly indicating the identity of the talker), though this may come at the cost of experimental control over the acoustic stimuli.

In addition, future research may investigate the *source* of these context and talker effects, specifically with respect to where in the auditory stream one may first begin to see these contrastive perceptual effects. For instance, a follow-up experiment implementing a version of Experiment 2a with high-pass filtered test stimuli (i.e., such that the F0 is missing in the signal) could establish whether the contrastive F0 context effects reported in the present study were primarily caused by the acoustic signal (i.e., the F0 itself) or by its psychoacoustic counterpart (i.e., pitch). The relative size of the context and talker effects may be susceptible to further changes in methodology as well. For instance, a conceptual replication with a longer or shorter exposure duration may help determine the exact relationship between exposure duration and the size of a potential talker effect. We also acknowledge that we have not fully explored the acoustic manipulation space used in our designs. We opted for a “fair” design with matched manipulation sizes (i.e., 4 st for context and for talker), which we believe allowed a potential context F0 effect and a talker F0 effect to manifest at comparable odds. To further explore the cue-weighing mechanisms that underlie our results, future work may use different manipulation sizes across the two types of cues. For instance, a study that implements a smaller manipulation size for contextual cues (e.g., 2 st) than talker cues (e.g., 4 st) might reveal that, once a certain threshold in cue variation range is reached, a talker F0 effect may appear alongside a context F0 effect. In other words, asymmetrically manipulating the manipulation sizes themselves may uncover whether variability translates to informativity when cues from different sources are being integrated.

The present study had two objectives: To test whether listeners can use talker F0 information during fricative perception, and to investigate the interaction of an F0 context effect with this talker F0 effect. Overall, in the three experiments which utilized the entire eight-step continuum (i.e., Experiments 1, 2b, and 3), we observed virtually no difference in response proportions across context or talker F0 conditions in the first and last steps of the continuum. Thus, we found no evidence that contextual F0 information was used in the identification of these unambiguous phonemes. However, in the more ambiguous steps of our fricative continuum, context F0 significantly drove fricative identification. Similarly, and crucially, talker F0 information was only utilized when the contextual F0 information was not sufficient to identify the target fricative. Overall, our findings suggest a gated or hierarchical structure in the perceptual system whereby gradually increasing amounts of both signal-driven and knowledge-driven processes are utilized on an “as-needed” basis, potentially reweighed as a function of the perceptual system’s confidence in their cues.

## Declarations

### Open practices statement

All stimuli, data visualization and analysis scripts, and non-identifiable participant data have been made publicly available on the Open Science Framework at: https://osf.io/5j4ye/. None of the experiments were pre-registered.
